# Effects of *Trichoderma strigosellum* in *Eucalyptus urophylla* Development and Leaf-Cutting Ant Behavior

**DOI:** 10.3390/jof8010015

**Published:** 2021-12-27

**Authors:** Kamilla Otoni Marques Batista, Dayara Vieira Silva, Vitor L. Nascimento, Danival José de Souza

**Affiliations:** 1 Graduate Program in Plant Production, Universidade Federal do Tocantins—UFT, Gurupi 77410-530, Brazil; kamillaotoni@live.com (K.O.M.B.); dayaravieira@hotmail.com (D.V.S.); vitorlnasc@gmail.com (V.L.N.); 2 Biology Department, Universidade Federal de Lavras—UFLA, Lavras 37200-000, Brazil

**Keywords:** antagonistic fungi, endophytic colonization, herbivory deterrent, microbial control, pest management, symbiosis

## Abstract

Fungal endophytes can protect plants against herbivory and be used to control leaf-cutting ants. In this study, we aimed to evaluate the potential of endophytic colonization of *Eucalyptus urophylla* by three filamentous fungal species and their influence on the plant development and foraging behavior of *Atta sexdens*. The study design was completely randomized and comprised a factorial scheme of 4 × 3, three antagonistic fungal species (*Escovopsis* sp., *Metarhizium anisopliae*, and *Trichoderma strigosellum*) of the leaf-cutting ant, and one control and three inoculation methods (conidial suspension via foliar spray [FS] and soil drench [SD] inoculation, and seedlings inoculated with mycelium [SWM]). The SWM method allowed *T. strigosellum* to colonize all plant organs, and these plants exhibited higher height, leaf number, shoot dry mass, and total dry mass than the ones subjected to the other inoculation methods. The SWM method increased the plant height than the control plants and those inoculated with *Escovopsis* sp. and *M. anisopliae*. *Trichoderma strigosellum*, previously isolated from soil, colonized *E. urophylla* plants and positively influenced their development, as demonstrated by the SWM method. *Trichoderma strigosellum* promoted the increase in *E. urophylla* height compared with when the FS and SD methods were used (by 19.62% and 18.52%, respectively). Our results reveal that *A. sexdens* workers preferentially began cutting the leaves from plants not previously colonized by *T. strigosellum*. This behavior can be explained by modifications in the phenotypic traits of the eucalyptus leaves.

## 1. Introduction

The genus *Eucalyptus* accounts for 72% of all planted forests in Brazil [1]. Leaf-cutting ants are considered the main insect pests in these cultivated forests [2]. *Eucalyptus* spp. can be attacked at all stages of the crop cycle; therefore, pest control is critical in both the pre- and post-planting phases [3]. *Eucalyptus urophylla* is one of the main species cultivated in Brazil [4], and several studies have demonstrated the susceptibility of *E. urophylla* to attack by different leaf-cutting ant species [5,6]. Damage caused by leaf-cutting ants in cultivated forests results from the constant cutting of fresh plant material, which is used as a substrate by the symbiotic fungus of the leaf-cutting ants *Leucoagaricus gongylophorus*. In turn, this mutualistic symbiotic fungus is used by leaf-cutting ants as food for their larvae and queen.

In addition to the mutualistic fungi, leaf-cutting ants interact with several microorganisms, including yeasts [7], bacteria [8], and filamentous fungi, such as the parasitic and antagonistic fungal species *L. gongylophorus, Escovopsis* spp., and *Trichoderma* spp. [9,10] and the entomopathogenic fungi *Metarhizium anisopliae* and *Beauveria bassiana* [11,12,13]. The diversity of microorganisms inside the colonies is mostly linked to the substrate transported by the foraging workers into the nest for fungal cultivation [14,15]. The number of endophytic fungi associated with leaf-cutting ants is continuously increasing [15,16,17,18]. Meanwhile, interest in using microorganisms to control leaf-cutting ants has increased in the last decade in Brazil, mainly owing to the restrictions imposed by environmental certification agencies, such as the Forest Stewardship Council (FSC). Sulfluramid, one of the main active ingredients in toxic baits for leaf-cutter ant control, is included in Annex B of the Stockholm Convention on Persistent Organic Pollutants. Efforts to develop safer and environment-friendly control methods are the need-of-the-hour [19]. Entomopathogenic fungi and antagonists can be used as biological control strategies for leaf-cutter ants, thus meeting the criteria of environmental certification agencies. The fungus *Trichoderma harzianum* is pathogenic to larvae and pupae, whereas *Beauveria bassiana* causes faster and higher mortality rates of *A. sexdens* workers [20]. A mixture of *B. bassiana* and *Trichoderma lignorum* spores has also been used to control another leaf-cutting ant, *Atta cephalotes*, in the fields. Effective control of 90% of the nests was observed in the field phase after 60 days. However, this result was limited to nests smaller than 50 m^2^ [21].

Endophytic fungi colonize, adapt, and propagate within plants. They can be isolated from leaves, stems, and roots, and inhabit living plant tissues without damaging the plant [22]. The host may even benefit from the colonization of endophytic fungi because of the production of compounds that promote plant growth and act against other microorganisms and herbivores [23,24]. Thus, they can influence and mediate essential relationships in plant-insect interactions [22,23,24,25,26]. The positive effects of *Trichoderma* spp. on the growth of *Eucalyptus* plants are known [27]. However, its effects on herbivores attacking aerial parts are not well understood [28].

Therefore, in this study, we aimed to evaluate (i) the potential of endophytic colonization of *E. urophylla* seedlings by the antagonistic fungi of the leaf-cutting ants *Escovopsis* spp., *M. anisopliae*, and *Trichoderma strigosellum*; (ii) the influence of these inoculated microorganisms on *E. urophylla* growth through biometric characteristics; and (iii) the cutting behavior and leaf consumption of leaf-cutting ants, to understand the mechanism of possible changes in this behavior after endophytic colonization by fungi.

## 2. Materials and Methods

### 2.1. Fungal Isolates and Monosporic Culture Preparation

The experiment was conducted at the Universidade Federal do Tocantins (UFT)—Gurupi campus in the northern region of Brazil, a Cerrado *stricto sensu* area. The three fungal species were isolated and identified at the Laboratory of Symbiosis Insects-Microorganisms between 2015 and 2017. *Escovopsis* sp. (ESC 001) was isolated from a colony of *Acromyrmex balzani* in the municipality of Gurupi, Tocantins State, and deposited in the UNESP-Microbial Resources Center (CRM-UNESP) with the code LESF 110. *Metarhizium anisopliae* (TCD 008) was isolated from a larva of *Tenebrio molitor* in contact with the forest soil of the UFT-Gurupi campus, using the bait technique (CRM 1397, GenBank: KX451122). *Trichoderma strigosellum* (TCD 003) was obtained from a soil sample from the UFT, Gurupi campus (GenBank: KU873087) [9].

Fungal inocula were prepared from monosporic cultures of the three species, according to Fernandez [29]. Pure colonies, developed on potato dextrose agar (PDA) supplemented with chloramphenicol (250 mg∙L^−1^), were incubated for seven days in Petri dishes (*T. strigosellum*) or 15 (*Escovopsis* sp. and *M. anisopliae*) days. Conidia were harvested with a flame-sterilized nichrome needle, suspended in a microtube with 1 mL of distilled water and sterile adhesive spreader (Tween 80 0.1% [*v*/*v*]), and vortexed for 10 s. Then, 100 μL of this suspension was added to Petri dishes containing agar-water medium and spread with a Drigalski spatula. Petri dishes were wrapped with Parafilm^®^ and incubated at 25 ± 2 °C with a 12-h photoperiod until the initiation of conidial germination. After germination, single isolated conidium was transferred to another Petri dish containing PDA supplemented with chloramphenicol (250 mg∙L^−1^). Wrapped Petri dishes were incubated in a climatic chamber at 25 ± 2 °C with a 12-h photoperiod for fungal development.

### 2.2. Inoculum Preparation

**Figure 1:**
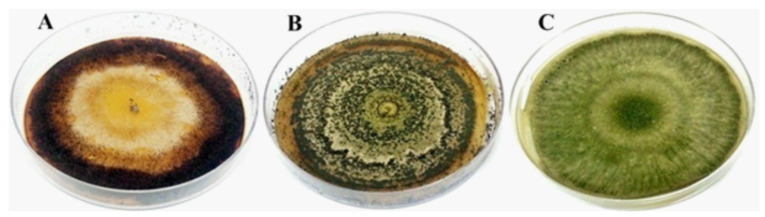
Fungal isolates. (**A**): *Escovopsis* sp.; (**B**): *Metarhizium anisopliae*; (**C**): *Trichoderma strigosellum*.

The inocula were obtained from pure colonies (Figure 1). Sterile distilled water with 0.1% (*v*/*v*) Tween 80 was added to the plate and gently scraped to obtain the conidial suspension under aseptic conditions in a flow chamber. This suspension was filtered through a triple layer of sterile gauze to retain mycelial fragments and remnants of the culture medium. Necessary dilutions were performed to quantify conidia. Conidial counting was performed using a Neubauer chamber, an optical microscope, and a manual counter. The concentration of each inoculum was adjusted to 1 × 10^8^ conidia·mL^−1^.

### 2.3. Eucalyptus Seedling Production for Foliar Spray and Soil Drench Inoculation

A total of 120 *Eucalyptus urophylla* seedlings were raised in polythene bags (12.5 × 12.5 cm); of them, 60 were used for foliar spray (FS) and 60 for soil drench (SD) inoculation. The commercial substrate used, Bioflora^®^ (Prata, Brazil) (coconut fiber, sphagnum peat moss, and other stable ingredients), was autoclaved at 120 °C for 30 min to avoid contamination. Five seeds of *E. urophylla*, LCFA 013 cultivar, from Sementes Caiçara LTDA (Brejo Alegre, Brazil), were sown in each bag. Selective thinning was conducted after 30 days, leaving only the more vigorous seedlings. Plants were watered daily until the day of evaluation and 12 weeks after germination. The plants were placed in a rectangular PVC cage (0.8 m × 1.0 m × 2.0 m), protected by cheesecloth to prevent insect attacking. The cages were kept in a nursery house covered with polythene under natural conditions. FS and SD inoculations were performed eight weeks after sowing.

FS and SD inoculations were used to inoculate the leaves and roots, respectively, according to the methodology described by Parsa et al. [29] with minor modifications. For the FS method, leaves were cleaned, and conidial suspensions of one of the three fungal species (treatments) or 0.1% Tween 80 (control) were applied to the adaxial surface of leaves with a standard atomizer, positioned at 5–8 cm in the intermediate pair of each plant until saturation was achieved. The top of the plastic bag was covered with aluminum foil to prevent conidial drainage of the soil. For the SD inoculation method, a graduated cylinder was used to apply 10 mL of the conidial suspension (treatments) or 0.1% Tween 80 (control) to the surface of the soil at the plant base. After inoculation, the plants were covered with a transparent plastic bag and incubated in a nursery house covered with polythene under natural conditions. The plants were covered for 48 h to maintain high air humidity.

### 2.4. Inoculation of E. urophylla Seedlings with Mycelium

As described for FS and SD inoculation, to treat seedlings with mycelium (SWM), *E. urophylla* seeds were surface sterilized with 1% sodium hypochlorite (NaClO) solution for 10 min and rinsed three times with sterile distilled water. Then, 75 seeds were distributed in four Petri dishes with agar-water medium, which were then wrapped with Parafilm^®^ and incubated at 25 ± 2 °C with a 12-h photoperiod. Seven days after seed germination, the 15 most vigorous seedlings from each plate were individually inoculated with a flame-sterilized nichrome needle containing hyphae from the three-day culture of *T. strigosellum*, or the seven-day cultures of *Escovopsis* sp. or *M. anisopliae*. Small wounds were made on the seedling stem with the needle under a stereoscopic microscope, thus allowing the fungus to come into contact with the internal seedling tissue. The control consisted of seedlings wounded but not inoculated with any of the fungal species. After inoculation, the plates were sealed again with Parafilm^®^ and incubated in a climatic chamber at 25 ± 2 °C with a 12-h photoperiod. Seven days after inoculation, the seedlings were transferred to polythene bags with an autoclaved substrate. The seedlings were watered daily until the day of evaluation, which was performed 12 weeks after germination.

### 2.5. Evaluation of Endophytic Colonization in Different Plant Organs

Stems, leaves, and roots were collected from entire seedlings separated from the substrate to evaluate endophytic colonization, according to the methodology employed by Rocha et al. [17] and Parsa et al. [30]. The re-isolated endophytes were identified using a dichotomous key for fungi and by culture comparisons with those of previously identified samples. For seedlings treated with FS, the pair of inoculated leaves was used as a reference, and a pair of leaves 5 cm above the stem and another pair of leaves 5 cm below the reference were collected. From the plants grown from the seedlings inoculated via SD, the first two pairs of leaves and two pieces of stems (5 cm each) were collected. For SWM, four leaves were randomly collected, and two fragments of 5 cm of the stem were removed from the middle part of the plant.

For all treatments, the roots were washed with tap water, and a piece was collected in the middle of the primary root, 1 cm underground, after the end of the stem. Collection as well as washing were performed under laminar flow and aseptic conditions. One 3.0-cm^2^ piece was cut out from each leaf and sequentially surface-sterilized in ethanol (70%, 1 min) and NaClO (1%, 4 min), then washed three times in sterile distilled water. Each fragment was cut into three smaller fragments (0.5 cm^2^). Similarly, the stem and root pieces were sequentially surface-sterilized. Each stem and root piece was then dissected into three 0.5 cm length sections (discarding the ends). Each section was cut longitudinally in the middle.

Six fragments from each pair of leaves and six fragments from each stem and root were placed in individual Petri dishes containing PDA supplemented with chloramphenicol (250 mg·L^−1^). All Petri dishes were sealed and incubated at 25 ± 2 °C with a 12 h photoperiod and were inspected for 20 days in the presence of *T. strigosellum*, *Escovopsis* sp., or *M. anisopliae*.

### 2.6. Assessment of Plant Biometric Characteristics

The following biometric characteristics were measured to analyze the effects of the presence of fungi on plant growth post-inoculation: plant height, number of leaves, shoot dry mass, root dry mass, and total dry mass. Plant height (cm) was measured from the base to the apex of the plants. Shoots were cut, and the roots were separated and washed under running tap water. Both the cut parts of the shoots and the separated roots were packed in paper bags and dried in a forced air circulation oven at 65 °C for 72 h to obtain the dry mass (g) of each plant part.

In addition, the treatments with the best results regarding endophytic colonization and biometric parameters were subjected to cutting preference trials by leaf-cutting ants. Leaf thickness (µm) was measured from a photomicrograph (10× magnification) of leaf sections using ImageJ software [31]. The color of leaves was quantified based on percentage per area, according to the method described by Schaberg et al. [32].

### 2.7. Maintenance of Atta Sexdens Colonies and Cut Behavior Assessment

The *A. sexdens* colonies used in this experiment, approximately two years old, were developed from fertilized females collected at UFT—Gurupi campus (−11.744085, −49.048808) and adapted to controlled laboratory conditions (25 ± 2 °C, 75 ± 5% relative humidity, 12 h photoperiod). Leaves of *Acalypha indica* L., *Mangifera indica* L., *Anacardium occidentale* L., *Citrus* sp., oat flakes, and wheat bran were used to feed the colonies.

For the cut preference trials, the colonies did not receive any food 24 h before the experimental procedures. Following the method described by Marsaro Júnior et al. [33]), whole leaves (approximately 0.2 g of the apical third of each plant) were collected and marked with enamel markers (Testors^®^—Rockford, IL, USA) to differentiate the plants containing the fungus from the controls. Based on our experience and the findings of previous studies, no adverse survivorship or behavioral effects from marking have been observed [34]. Each leaf was placed in a Petri dish, weighed on a semi-analytical balance, and placed simultaneously inside each colony for 30 min. During this time, we observed that the treatment leaves were the first to be cut and transported into the colony. The unloaded leaf fragments were then weighed again, and their consumption was calculated based on the weight difference. At the same time, during the test, a leaf of approximately equal weight was placed next to the colonies. This leaf was weighed at the beginning and end of each repetition in order to calculate the water loss that occurred during the experimental procedures.

### 2.8. Experimental Design and Statistical Analysis

The design was completely randomized using a 4 × 3 factorial scheme with 15 replicates. Each plot consisted of one seedling. The factors included three fungal species (*Escovopsis* sp., *M. anisopliae*, and *T. strigosellum*) plus one control and three inoculation methods (FS, SD, and SWM). Ten seedlings were used for the evaluation of endophytic colonization, and five seedlings were used to evaluate the biometric characteristics (plant height, number of leaves, shoot dry mass, root dry mass, total dry mass, and leaf thickness). The data were subjected to analysis of variance (ANOVA) with the F-test, followed by Tukey’s test when appropriate; *p* < 0.05, was considered statistically significant, using SISVAR^®^ 5.7 software (Lavras, Brazil) [35]. For the cut behavior assessment, the data obtained were analyzed using the Shapiro–Wilk normality test, with log transformation at base 10 when necessary. For the first cut evaluation, the test for determining the difference between two proportions was performed using Statistica^®^ 7.1 software (Palo Alto, CA, USA).

## 3. Results

### 3.1. Endophytic Colonization

**Figure 2:**
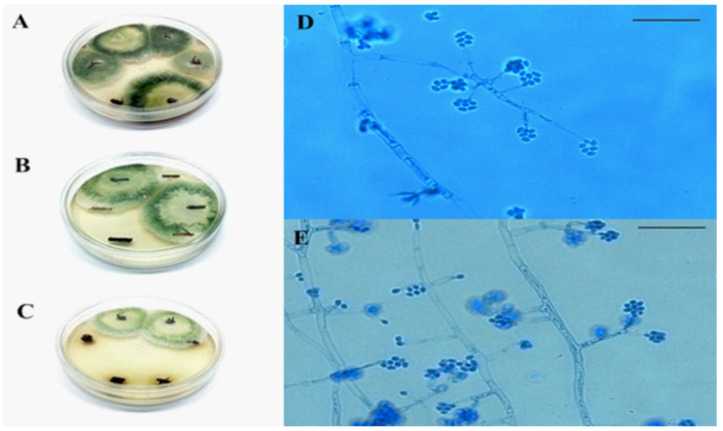
Endophytic colonization of *Eucalyptus urophylla* by *Trichoderma strigosellum* in (**A**): five root sections in the seedling inoculation method; (**B**): two stem sections in the soil inoculation method; (**C**): two sections of eucalyptus roots in the soil inoculation method; (**D**,**E**): conidia and conidiophores of the endophyte *T. strigosellum* visualized under an optical microscope, magnified 400×. The scale bar corresponds to 10 µm.

*Trichoderma strigosellum* was the only fungus capable of endophytically colonizing *E. urophylla* in the SWM and SD inoculation methods (Table 1). In the SWM method, this fungus was detected in 90% of the roots, 70% of the stems, and 60% of the leaves of the treated plants. Using the SD method, *T. strigosellum* was detected in 50% of the roots and 60% of the stems of the treated plants. In contrast, *Escovopsis* sp. and *M. anisopliae* were not detected in *E. urophylla* plants, regardless of the inoculation method (Table 1, Figure 2). Moreover, seedlings inoculated with *Escovopsis* sp. mycelium (SWM) showed 100% mortality. There were three consecutive attempts to inoculate the seedlings with this method; however, the results were the same each time.

### 3.2. Biometric Characteristics

A significant difference was found only in the plant height with respect to the inoculation method. For plant height, number of leaves, shoot, and total dry mass, the fungal species factor differed significantly. There was no difference in the root dry mass between the two factors (fungal species and inoculation method). The interaction effect of the factors, inoculation methods and fungal species, was observed for all the evaluated traits (Table 2), and therefore, the data were analyzed considering the interaction between factors.

**Figure 3:**
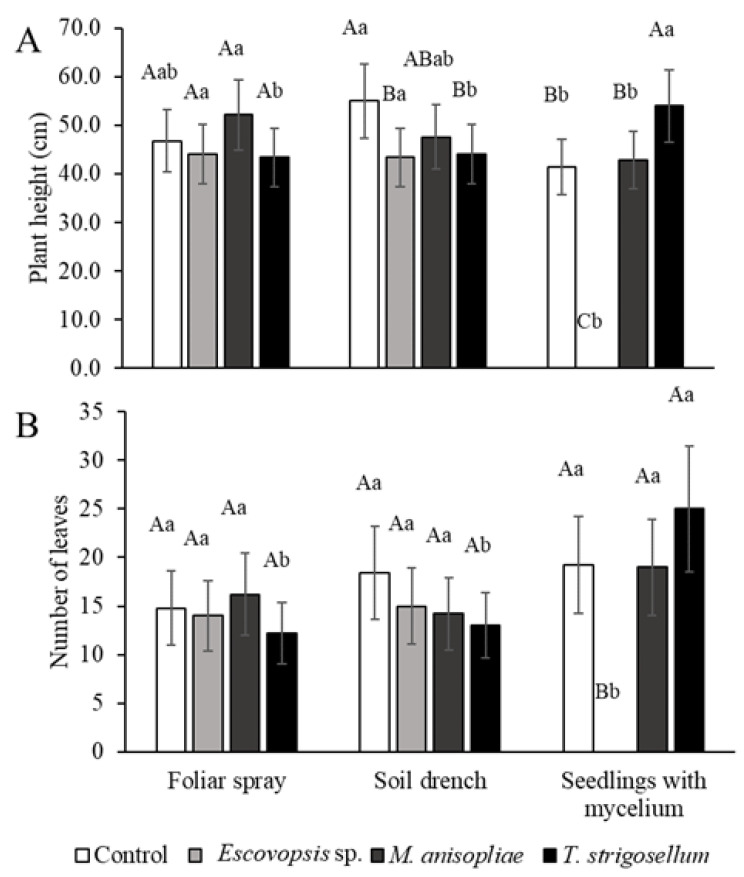
Biometric characteristics of *E. urophylla* in response to inoculation methods (Foliar spray, soil drench, and seedlings with mycelium) and inoculated fungal species (*Escovopsis* sp., *Metarhizium anisopliae*, and *Trichoderma strigosellum*). (**A**) Plant height (cm), and (**B**) Number of leaves per plant. Mean values with the same uppercase letter indicating the effects of the fungi or lowercase letter indicating the effects of the methods did not differ, as per Tukey test (*p* < 0.05). Each column corresponds to the mean of five plants ± standard error (SE).

Plant height was lower in the SWM method than in the other methods, except for the *T. strigosellum* treatment (Figure 3A). *Eucalyptus urophylla* plants inoculated with *Escovopsis* sp. did not differ from control plants for any biometric trait, independent of the method (Figure 3A), with the exception of the SWM once this fungus in this treatment was lethal. The plant height post *M. anisopliae* treatment did not differ from that observed post the other treatments, for any of the factors analyzed (Figure 3A). The control plants in the SD method were taller than those in the SWM method and those inoculated with *Escovopsis* sp. and *T. strigosellum* (Figure 3A). Higher height values were also observed in plants inoculated with *M. anisopliae* in the FS method, according to the SWM method (Figure 3A). There was an increase in the number of leaves (Figure 3B) in the SWM in the *T. strigosellum* treatment.

**Figure 4:**
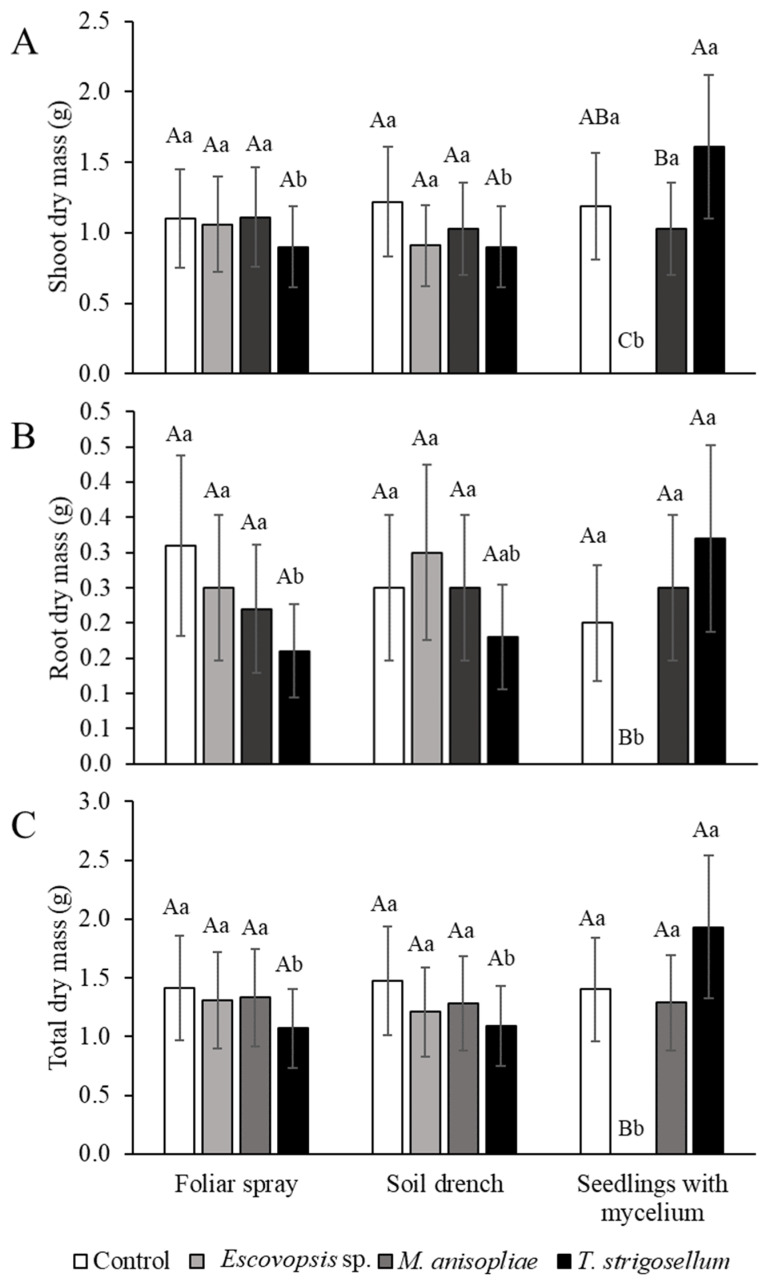
Biometric characteristics of *Eucalyptus urophylla* in response to inoculation methods (Foliar spray, soil drench, and seedlings with mycelium) and inoculated fungal species (*Escovopsis* sp., *Metarhizium anisopliae*, and *Trichoderma strigosellum*). (**A**) Shoot dry mass (g), (**B**) Root dry mass (g), and (**C**) Total dry mass (g). Mean values with the same uppercase letter indicating the effects of the fungi or lowercase letter indicating the effects of the methods did not differ, as per Tukey test (*p* < 0.05). Each column corresponds to the mean of five plants ± standard errors (SE).

In relation to dry biomass, shoot, root, and total dry mass were highly influenced by the method. However, no significant differences were observed between groups (Figure 4).

### 3.3. Foraging Behavior

**Figure 5:**
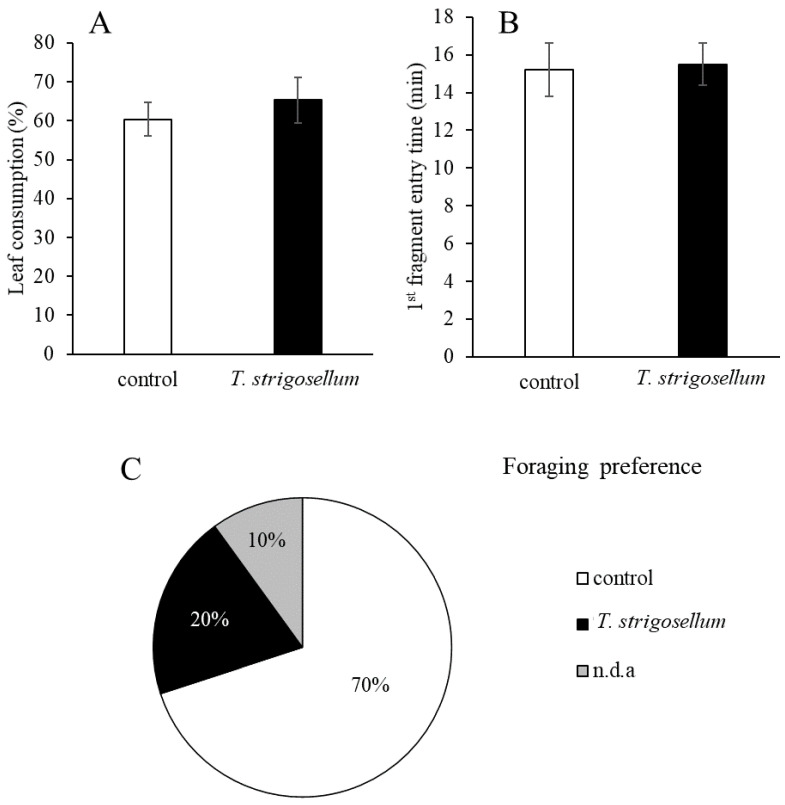
Changes in cutting preference of *Atta sexdens* for *Eucalyptus urophylla* inoculated with *Trichoderma strigosellum*. (**A**) Leaf consumption (%), (**B**) Time to entry in the first fragment of leaves (min), each column corresponds to the mean of 10 leaves ± standard errors (SE); (**C**) Foraging preference (%), indicating that in many colonies (70%) workers began cutting the leaves of the control group; proportions were significantly different (*p* < 0.05).

Leaf-cutting ants consumed 65.31% and 60.33% of the leaves of *E. urophylla* with and without *T. strigosellum*, respectively; however, the difference was not statistically significant (Figure 5A). Moreover, the difference in the foraging time between the treatments (control and *T. strigosellum* with SWM inoculation) was less than one minute (Figure 5B). Regarding the foraging behavior of the leaf-cutting ants, of the 10 colonies evaluated, ants from seven colonies began cutting the leaves of the control (without *T. strigosellum*), two began cutting leaves with the fungus, and one did not cut leaves from any of the treatment groups (Figure 5C).

### 3.4. Mechanisms of Plant Defense

**Figure 6:**
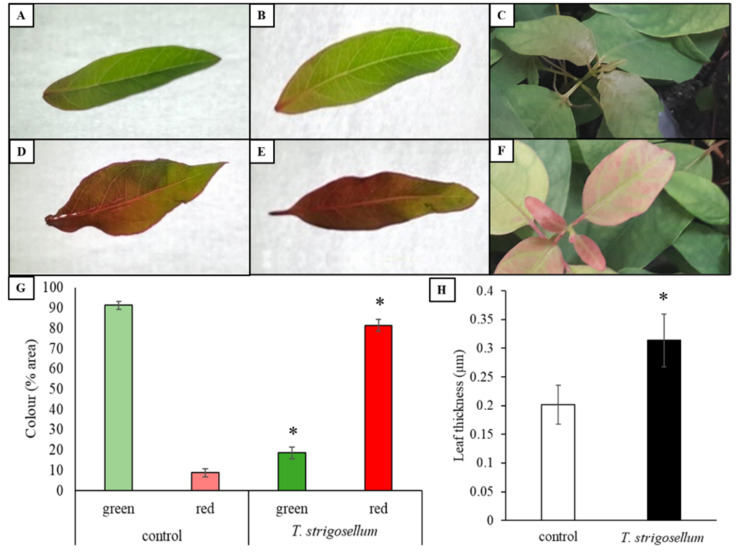
Changes in leaf color of *Eucalyptus urophylla* inoculated with *Trichoderma strigosellum*. Control (**A**–**C**) and *T. strigosellum* treatment (**D**–**F**). (**G**) Quantification of the relative leaf area spectrally identified as green and red in leaves of *E. urophylla*. Asterisks (*) in *T. strigosellum* treatment indicate values determined by the Tukey test to be significantly different (*p* < 0.05) from the control. Each column corresponds to the mean of eight leaves ± standard error (SE); (**H**) leaf thickness (µm) of *E. urophylla* inoculated with *T. strigosellum*. Asterisks (*) in *T. strigosellum* treatment indicate values determined by the Tukey test to be significantly different (*p* < 0.05) from the control. Each column corresponds to the mean of five plants ± standard error (SE).

To understand this change in the behavior of leaf-cutting ants, we analyzed the variation in the color and thickness of the leaves (Figure 6). Control plants mostly had green leaves (Figure 6A–C, 4 g), while *E. urophylla* inoculated with *T. strigosellum* had reddish leaves (Figure 6D–F, 4 g). Furthermore, *E. urophylla* plants inoculated with *T. strigosellum* had thicker leaves than those of the control (without inoculation) (Figure 6H).

## 4. Discussion

*Trichoderma strigosellum* is the only fungus tested here that can colonize all tissues of *E. urophylla*, and despite its limited effects on plant growth, it affected the cutting preference of and leaf consumption by leaf-cutting ants by increasing leaf thickness and changing leaf color (reddish leaves). In the SWM method, *T. strigosellum* promoted an increase in the height of *E. urophylla*, ranging from 19.62% to 18.52%, compared with that observed in the FS and SD methods. The FS method increased the number of leaves by 51.2 %, while the SD method increased the number of leaves by 48%. A 44.1% increase in shoot dry matter was observed compared with that in the other two methods. The SWM method increased the total dry matter by 44.56% and 43.53% compared with the FS and SD inoculation methods, respectively. For root dry matter, the difference was about 50% between SWM and FS methods. Similar results have been reported previously: inoculation of seeds with *Trichoderma virens* increased seedling height, shoot fresh mass, and total fresh mass of passionfruit plants, whereas the FS method did not produce any of these changes [36]. Researchers who studied the inoculation method and the emergence of plant diseases also observed that the inoculation method affected the time of onset and severity of the disease in the *Diaporthe* fungi–soybean plant system [37]. According to these authors, the pathogen recovery for *Diaporthe aspalathi* was the highest in plants inoculated with the stem-wound and toothpick methods compared with that achieved using less invasive methods, such as spore injection and mycelium contact.

Fungal recovery mainly from the roots of SWM-treated plants indicated that the mycelium of *T. strigosellum* was oriented downward from the inoculated site. By contrast, the SD method allowed upward colonization, which was limited to the stem. Other studies have verified that different *Trichoderma* species colonized all the organs of *Passiflora edulis* and *Theobroma cacao* plants [36,38]. However, the interaction between *Trichoderma* spp. and plants occurs mainly in the roots [39]. For instance, different isolates of *Trichoderma* have been detected only in the seedling roots of *Eucalyptus* spp. after using different inoculation methods [40,41]. Here, the FS method did not allow *T. strigosellum* to colonize the plant.

The interaction between plants and endophytic microorganisms is complex [42]. Several factors, such as host species and cultivar, endophyte species and strain, inoculum concentration or method, developmental phase of the host plant, and abiotic conditions can influence the artificial introduction of entomopathogenic endophytes [30,43,44,45]. *Trichoderma strigosellum* has been isolated from an incipient nest of *A. sexdens* [9], and in the laboratory conditions assay, the fungus inhibited the mutualistic fungus colonizing the leaf-cutting ants. Its inoculation in *Eucalyptus* plants is promising for a “Trojan-Horse” strategy to reduce the negative impact of these insects on *Eucalyptus* culture [17].

Our results potentially disprove the hypothesis that an endophytic stage of *Escovopsis* spp. might occur within the leaves that ants collect [25]. Further studies on the mechanisms used by *Escovopsis* to kill *Eucalyptus* plants are needed to gain insights into the parasite’s life cycle. Studies have pointed out that *Escovopsis* is not only lethal to the ant-targeted cultivar *L. gongylophorus*, but its secretions can also kill worker ants [46].

*Eucalyptus urophylla* oil was more fungitoxic than *Eucalyptus citriodora* and *Eucalyptus camaldulensis* essential oils [47]. According to Coley and Barone [48], antifungal substances, which are more prominent in young leaves, could have hindered endophytic colonization in the FS method. Therefore, it is fundamental to determine the suitable fungal and host plant species, as well as the best method of inoculation, to obtain reliable results. The plants inoculated with *Escovopsis* sp. were also influenced by the SWM method, but the overall effect was negative because, in this method, the fungus caused seedling death.

*Trichoderma strigosellum* colonized endophytic *E. urophylla* seedlings using the SD method. However, there was no increase in any of the biometric characteristics evaluated for these plants. Another study concluded that *T. virens* did not produce promising results in sugarcane plants because the fungus was unable to promote their growth [49]. Several factors, such as crop type and developmental conditions, limit plant growth and development in response to endophytic colonization, resulting in highly variable results [50,51]. According to Harman et al. [52], better development of plants can be achieved when *Trichoderma* is inoculated under stress conditions. The potential of *T. harzianum* isolate T-22 to promote plant growth and development is linked to its ability to solubilize relevant plant nutrients [53]. Other mechanisms that are linked to the ability of *Trichoderma* species to promote plant growth include the production of compounds that induce growth and control of secondary pathogens that can slow root growth and activity [54].

Endophytic fungi can alter the biochemical characteristics of the leaves, indicating that they can negatively influence the mutualistic fungi of leaf-cutting ants by affecting the foraging preference of the ants [55]. Changes in the leaf color can be directly associated with protection against herbivory since reddish colors can act as warning signals to insects [56,57,58]. We observed biochemical differences between leaves with and without *T. strigosellum*, with the former showing reddish leaves (Figure 6) and explaining the difference in the foraging behavior (Figure 5). Another important trait related to plant defense against herbivory is leaf thickness [59,60,61,62], which can influence the cutting behavior of leaf-cutting ants. Endophytic fungi can cause morphological and mechanical changes, ranging from an increase in the cell wall thickness to an increase in cuticle thickness [60]. Here, we found that leaves inoculated with *T. strigosellum* were thicker than leaves without inoculation (Figure 6). It is important to highlight that we found two possible mechanisms of plant defense via biochemical and mechanical changes, which are distinct from those described by Muiruri et al. [59] and Chen et al. [62]. Because of the various factors that may interfere with the relationship between fungi and plants, such as the host plant, we intend to continue this study in other species and plant families to obtain better results.

*Trichoderma strigosellum* promotes the development of eucalyptus seedlings and affects the behavior of an important insect pest in the Americas. The endogenous mechanisms underlying these effects deserve further investigation, considering that *Trichoderma* strains may be of particular relevance in *Eucalyptus* culture.

## Figures and Tables

**Table 1:** Re-isolation of the fungal species (*Escovopsis* sp., *Metarhizium anisopliae*, and *Trichoderma strigosellum*) inoculated using three methods (foliar spray, soil drench, and seedlings with mycelium) in the leaves, stems, and roots of *Eucalyptus urophylla* (*n* = 10).

Inoculation Methods	Fungi	Plant Organs		
		Leaves	Stem	Roots
Foliar spray	Control	0	0	0
	*Escovopsis* sp.	0	0	0
	*Metarhizium anisopliae*	0	0	0
	*Trichoderma strigosellum*	0	0	0
Soil drench	Control	0	0	0
	*Escovopsis* sp.	0	0	0
	*Metarhizium anisopliae*	0	0	0
	*Trichoderma strigosellum*	0	60%	50%
Seedlings with mycelium	Control	0	0	0
	*Escovopsis* sp.	-	-	-
	*Metarhizium anisopliae*	0	0	0
	*Trichoderma strigosellum*	60%	70%	90%

**Table 2:** Summary of the analysis of variance for plant height, number of leaves, shoot dry mass, root dry mass, and total dry mass of *Eucalyptus urophylla*, according to the inoculation methods (foliar spray, soil drench, and seedlings with mycelium) and inoculated fungal species (*Escovopsis* sp., *Metarhizium* *anisopliae*, and *Trichoderma strigosellum*).

Traits	Source of Variation				Mean	C.V. (%)
	Inoculation Methods (M)	Fungal Species (F)	M × F	Residual		
	Degrees of Freedom					
	2	3	6	48		
Plant height	1045.71 *	1261.35 *	886.58 *	35.15	42.88	13.83
Number of leaves	11.31 ^ns^	198.28 *	217.98 *	15.4	15.08	26.02
Shoot dry mass	0.03 ^ns^	0.85 *	0.82 *	0.1	1.00	31.77
Root dry mass	0.01 ^ns^	0.01 ^ns^	0.05 *	0	0.22	41.34
Total dry mass	0.09 ^ns^	1.06 *	1.26 *	0.15	1.23	31.50

* significant at 5% probability (*p* ≤ 0.05); ^ns^ not significant (*p* ≥ 0.05) by the *t*-test.

## Data Availability

The datasets used and/or analyzed during the current study are available from the corresponding author on reasonable request.
